# Nonlocal contrast calculated by the second order visual mechanisms and its significance in identifying facial emotions

**DOI:** 10.12688/f1000research.28396.1

**Published:** 2021-04-06

**Authors:** Vitaly V. Babenko, Denis V. Yavna, Pavel N. Ermakov, Polina V. Anokhina

**Affiliations:** 1 Department of Psychophysiology and Clinical Psychology, Academy of Psychology and Education Sciences, Southern Federal University, Rostov-on-Don, Russian Federation

**Keywords:** face, emotion, saliency, spatial heterogeneity, nonlocal contrast, second-order visual mechanisms

## Abstract

**Background:** Previously obtained results indicate that faces are preattentively detected in the visual scene, and information on facial expression is rapidly extracted at the lower levels of the visual system. At the same time different facial attributes make different contributions in facial expression recognition. However, it is known, among the preattentive mechanisms there are none that would be selective for certain facial features, such as eyes or mouth.

The aim of our study was to identify a candidate for the role of such a mechanism. Our assumption was that the most informative areas of the image are those characterized by spatial heterogeneity, particularly with nonlocal contrast changes. These areas may be identified in the human visual system by the second-order visual mechanisms selective to contrast modulations of brightness gradients.

**Methods:** We developed a software program imitating the operation of these mechanisms and finding areas of contrast heterogeneity in the image. Using this program, we extracted areas with maximum, minimum and medium contrast modulation amplitudes from the initial face images, then we used these to make three variants of one and the same face. The faces were demonstrated to the observers along with other objects synthesized the same way. The participants had to identify faces and define facial emotional expressions.

**Results:** It was found that the greater is the contrast modulation amplitude of the areas shaping the face, the more precisely the emotion is identified.

**Conclusions:** The results suggest that areas with a greater increase in nonlocal contrast are more informative in facial images, and the second-order visual mechanisms can claim the role of filters that detect areas of interest, attract visual attention and are windows through which subsequent levels of visual processing receive valuable information.

## Introduction

Experiments involving a saccadic task ( Crouzet *et al.*, 2010; Kirchner & Thorpe, 2006) and registration of event-related potentials ( Cauchoix *et al.*, 2014; Dering *et al.*, 2011; Herrmann *et al.*, 2005; Liu *et al.*, 2002; Liu *et al.*, 2009; Pitcher *et al.*, 2007) showed that face identification and processing are so fast that we can most probably speak here of a feedforward processing directed by preattentive mechanisms ( Crouzet & Thorpe, 2011; Reddy *et al.*, 2004; Reddy *et al.*, 2006; VanRullen, 2006; Vuilleumier, 2000; Vuilleumier, 2002; Vuilleumier *et al.*, 2001), that is without any involvement of attention ( Allen *et al.*, 2017; Eimer & Holmes, 2002; Liu *et al.*, 2002). This may mean that low level information is used to distinguish a face from the background and to define its characteristics.

Many researchers believe that faces are holistically coded within the low-frequency range, and this description is sufficient not just to detect the face but also to determine its emotional expression ( Calder *et al.*, 2000; Schyns & Oliva, 1999; Tanaka *et al.*, 2012; White, 2000). Meanwhile the classical work by A.L. Yarbus (1967) clearly demonstrated that while viewing a face we fix our eyes at quite definite details. Further eye tracking experiments and experiments with the “bubbles” method showed that not all areas of the face are equally useful for emotion recognition ( Blais *et al.*, 2017; Duncan *et al.*, 2017). Different facial features are significant for the discrimination of different emotions ( Atkinson & Smithson, 2020; Calvo *et al.*, 2014; Eisenbarth & Alpers, 2011; Fiset *et al.*, 2017; Jack *et al.*, 2014; Smith & Merlusca, 2014; Smith & Schyns, 2009; Smith *et al.*, 2005; Wang *et al.*, 2011), these emotions being probably processed at different rates too ( Ruiz-Soler & Beltran, 2012).

The problem is that the lower levels of the human visual system lack neurons which would be selective to certain facial features. Nevertheless, there should exist a mechanism permitting the detection of faces automatically and to extract significant information preattentively. The aim of this investigation was to identify the possible candidate for the above mechanism.

Realization of the importance of defining those areas of interest in the images that attract visual attention, was the impetus for those research trends aimed at finding the algorithm of formation of saliency maps ( Borji *et al.*, 2013; Judd *et al.*, 2012; Rahman *et al.*, 2014). At the same time, the choice of the attention goal should be based on the principle of information maximization ( Bruce & Tsotsos, 2005).

In respect of the human visual system, one can only speak of the preattentive mechanisms actualized within low-level vision and able of “bottom-up” attention control. It is clear that the evenly lit areas are of no interest to the visual system. Of interest is something changeable, hence we may speak of changes in brightness. Indeed, there are specialized mechanisms for finding brightness gradients in the visual system, and these are striate neurons ( Hubel & Wiesel, 1962; Hubel & Wiesel, 1968). However, these can only find local heterogeneities. To find areas of interest, there should exist mechanisms beyond local operations. Yet we first have to answer the question about the characteristics of these nonlocal areas of interest. In recent years, there appeared a viewpoint stating that the image areas whose information content differs from the surroundings are of the greatest interest for the visual system ( Baldi & Itti, 2010; Hou *et al.*, 2013; Itti & Baldi, 2009). This refers to difference in low-level feature distribution in the field of view ( Itti *et al.*, 1998), while salience in this case is determined by the degree of total difference of features within the analyzed area from features in the surrounding area ( Bruce & Tsotsos, 2009; Gao & Vasconcelos, 2007; Perazzi *et al.*, 2012).

Important is that the visual system really has mechanisms able to find space heterogeneities of brightness gradients: these are the so-called second-order visual mechanisms (see review Graham, 2011). The latter combine the outputs of striate neurons, and their receptive fields are organized in such a way that they do not respond to homogeneous textures, but are activated when the texture has modulations of contrast, orientation, or spatial frequency of brightness gradients.

So far these mechanisms have been predominantly studied and considered as an instrument of segmentation of textures (e.g. Graham & Sutter, 2000; Graham & Wolfson, 2004; Kingdom *et al.*, 2003; Schofield & Yates, 2005). We are pioneers in raising the question whether the second-order visual filters can be of use in segmenting natural images and finding in them those saliency areas that are used for categorization. Our expectation was to obtain the answer through the task of detecting faces in a series of successively presented objects and determining their emotional expression.

It was shown earlier that the second-order mechanisms are specific to the modulated visual feature, i.e. whether it is contrast, orientation or spatial frequency of brightness gradients ( Babenko & Ermakov, 2015; Kingdom *et al.*, 2003). Then it was revealed that modulations of contrast take priority in competition for attention ( Babenko *et al.*, 2020). All this enabled us to work out a hypothesis stating that areas of maximum modulation of nonlocal contrast contain information helpful in identifying emotional facial expressions. To test this hypothesis, we developed a software program (gradient operator of nonlocal contrast) imitating operation of the second-order visual filters and calculating the space distribution of contrast modulation amplitude in the input image.

## Methods

A total of 38 students between the ages of 19 and 21 took part in this investigation. All the participants had normal or corrected to normal vision and reported no history of neurological or psychiatric disorders. All the research participants were informed about the purpose and procedures of the experiment; they all signed a consent form that outlined the risks and benefits of participating in the study and indicated that they believed in the safety of the investigation. The study was conducted in accordance with the ethical standards consistent with The Code of Ethics of the World Medical Association (Declaration of Helsinki) and approved by the local ethics committee. The design of the experiment, the methodological approach, the conditions of confidentiality and use of the consent of participants were performed according to the Code of Ethics of Southern Federal University (SFU; Rostov-on-Don, Russia) and approved ethically by the Academic Council of the Academy of Psychology and Pedagogy of SFU, on 25 March, 2020.

Initial digitized photos of faces and objects brought to a single size (8 ang.deg.), medium brightness (35 cd/m2) and RMS contrast (0.45), were processed by the nonlocal contrast gradient operator. A total energy of the image filtered at a frequency of 4 cycles per a diameter of this central area with a 1 octave bandwidth, was calculated in the center of the operator’s concentric area. In the peripheral part of the operator (a ring whose width equaled the central area diameter), the spectral power of the entire range of spatial frequencies perceived by a person was calculated, per 1 octave on average.

The contrast modulation amplitude amounted to the difference of values of the power spectrum obtained in the operator’s central and periphery areas. Operators of various diameters were used, and for each operator we defined those areas where the total contrast was maximum different from the surroundings, i.e. had the highest modulation amplitude.

**Figure 1.**
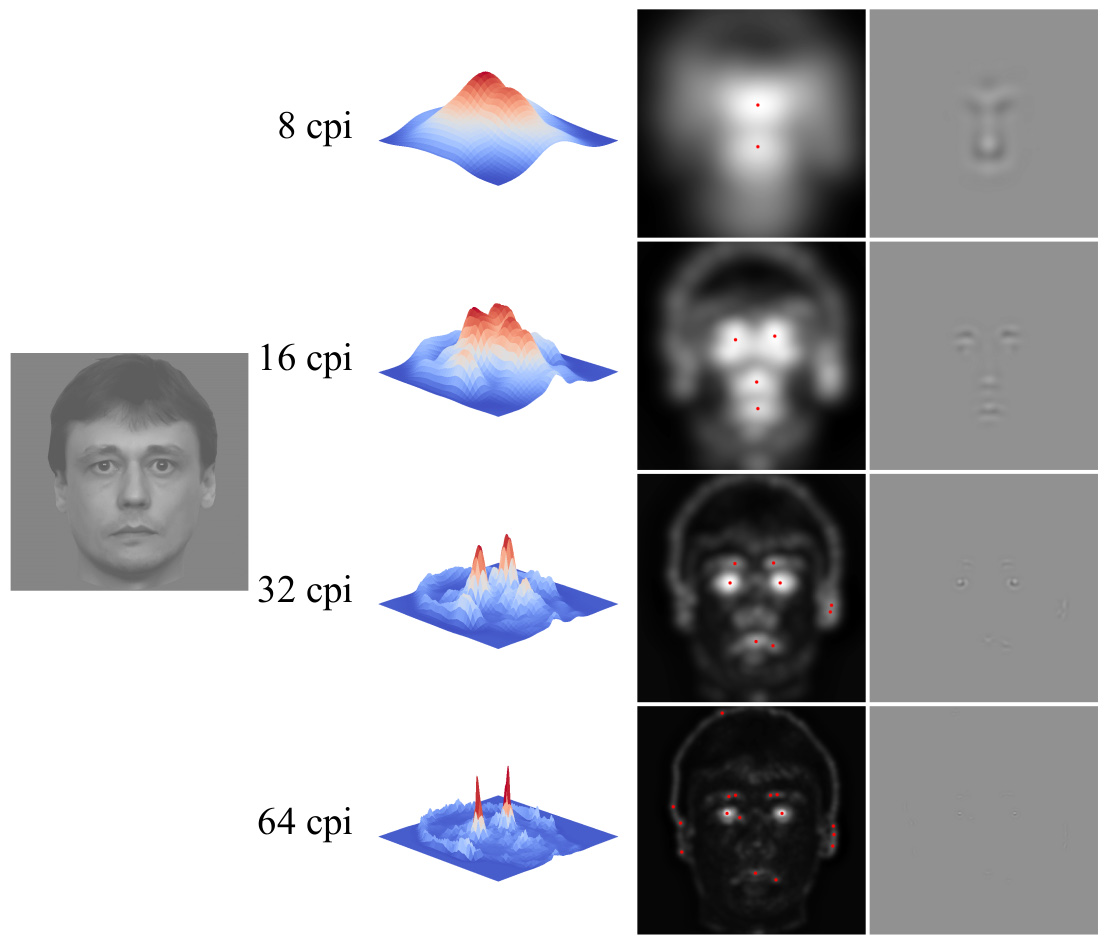
Algorithm of facial stimuli formation.

The algorithm of stimuli formation is shown in Figure 1. An initial image example can be seen on the left. Then there are spatial frequencies in cycles per image (cpi) for which space distribution of the total nonlocal contrast was defined. On the right, one may see 3D maps of space distribution of contrast modulation amplitude when using operators of various diameters. The next column demonstrates the same maps in a 2D format. Red dots on them show local maximum apexes. While processing the image with the gradient operator of the largest size with its central area diameter making one half of the image size, we selected 2 maximums, after which, in the course of operator diameter two-fold reduction, selected were 4, 8 and 16 maximums correspondingly. A round aperture with a Gaussian transfer function transmitting four image cycles (hereinafter this aperture is referred to as a “window”) was placed within positions found this way. Areas of maximum contrast modulation amplitude were combined in a new image (the right column). The total diameter of the areas found at different spatial frequencies equaled the diameter of a conventional circle with the initial image fit to it. Stimuli were the faces synthesized from the areas extracted at one spatial frequency (examples can be seen in the right column of Figure 1), as well as those resulting from the combination of these images within one aggregate image (i.e. containing all the previously used spatial frequency ranges).

To create stimuli, we used 40 initial images of faces and 240 initial images of natural objects. Photos of faces were taken from FERET Database collected under the FERET program, sponsored by the DOD Counterdrug Technology Development Program Office ( Phillips *et al.*, 1998; Phillips *et al.*, 2000). This database was created with the consent of participants and contains photographs of men and women of different races with different emotional facial expressions. We used part of the images from the database provided to us in full accordance with Color FERET Database Release Agreement.

In fact, we used the “bubbles” method ( Gosselin & Schyns, 2001), yet unlike the traditional approach with the aperture located at random, the aperture of our research was placed in definite, previously pre-estimated positions which corresponded to the areas with a definite modulation value of the total nonlocal contrast.

Then the same way we formed stimuli consisting of areas with the minimum contrast modulation amplitude, as well as images consisting of areas with a modulation having the medium amplitude between the closest minimums and maximums.

We employed a one-way design for independent samples having a three-level factor “Amplitude of modulation” (min, med, max). The percentage of correct identification of facial expressions was the dependent variable. The sample size was determined based on Anova's power = 0.8 and expected Cohen's f > 0.5 effect size. The minimum expected effect size was determined based on the results of the preview of the prepared images performed by the researchers themselves.

The observers were demonstrated synthesized images of Caucasian and Asian faces in frontal view (male and female) with neutral and happy facial expressions. These randomly alternated with synthesized images of objects of different categories, the probability of faces within the chains of consequent stimuli making 33%. The observer had to inform about the appearance of a face and possibly define its expression (the answer “I don’t know” was allowed). Exposure time was not limited. The percentage of correct recognitions of facial expressions for the images formed from the areas of different contrast modulation amplitudes, was calculated.

In order to anonymize the identity of the observers, all names were encrypted by md5 algorithm and raw data files were saved on the local disk storage with limited access.

## Results

**Figure 2.**
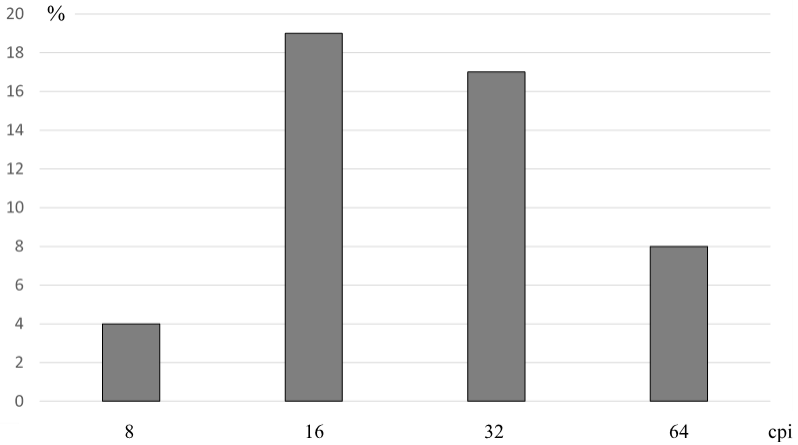
Comparison of the accuracy of distinguishing emotional expressions of faces collected from areas with maximum nonlocal contrast, containing different spatial frequencies. Axis X shows the central spatial frequency of the areas from which the face stimulus was synthesized.

First, we compared task solution effectiveness where the face images had been formed from maximum nonlocal contrast areas belonging to the narrow spatial frequency range. It is worth reminding that the lesser the diameter of the areas, the higher the spatial frequency (cpi) contained in them and the greater the general number of the areas found. Where synthesized face images contained space frequencies of just one range of 1 octave, the general result of facial expression recognition was low ( Figure 2). The performance was higher for the stimuli formed from the areas with the maximum increase in contrast having the central spatial frequency of 16 cpi. Somewhat lower were the values of 32 cpi frequency, and much lower these were for the lowest and the highest frequency ranges. The obtained distribution generally agrees with the data suggesting that the medium spatial frequency range expressed in cycles per face is more important in face recognition ( Boutet *et al.*, 2003; Collin *et al.*, 2006; Näsänen, 1999; Parker & Costen, 1999; Tanskanen *et al.*, 2005; see also review Ruiz-Soler & Beltran, 2006).

However, our main purpose was to test the hypothesis stating that the most informative image areas are those with the greatest increase in nonlocal contrast using the example of faces of different emotional expressions.

**Figure 3.**
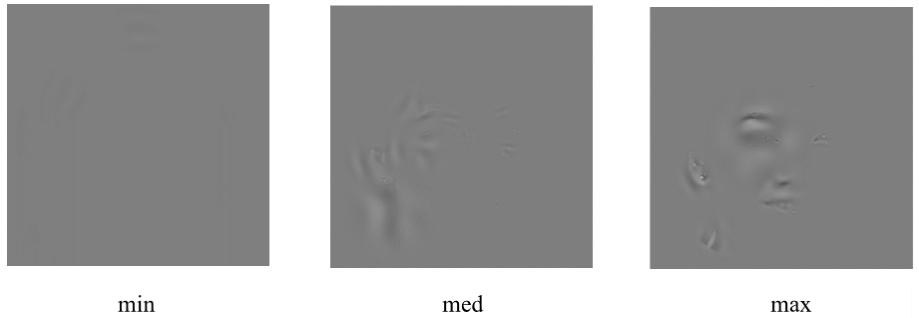
Examples of the face images formed from the areas of minimum (min), medium (med) and maximum (max) nonlocal contrast modulation amplitude.

To answer this question, we compared the effectiveness of task solution for the faces formed from the areas of different contrast modulation amplitudes: maximum, minimum and medium ( Figure 3). The stimuli were combined from the areas found in all the applied spatial frequency ranges.

**Figure 4.**
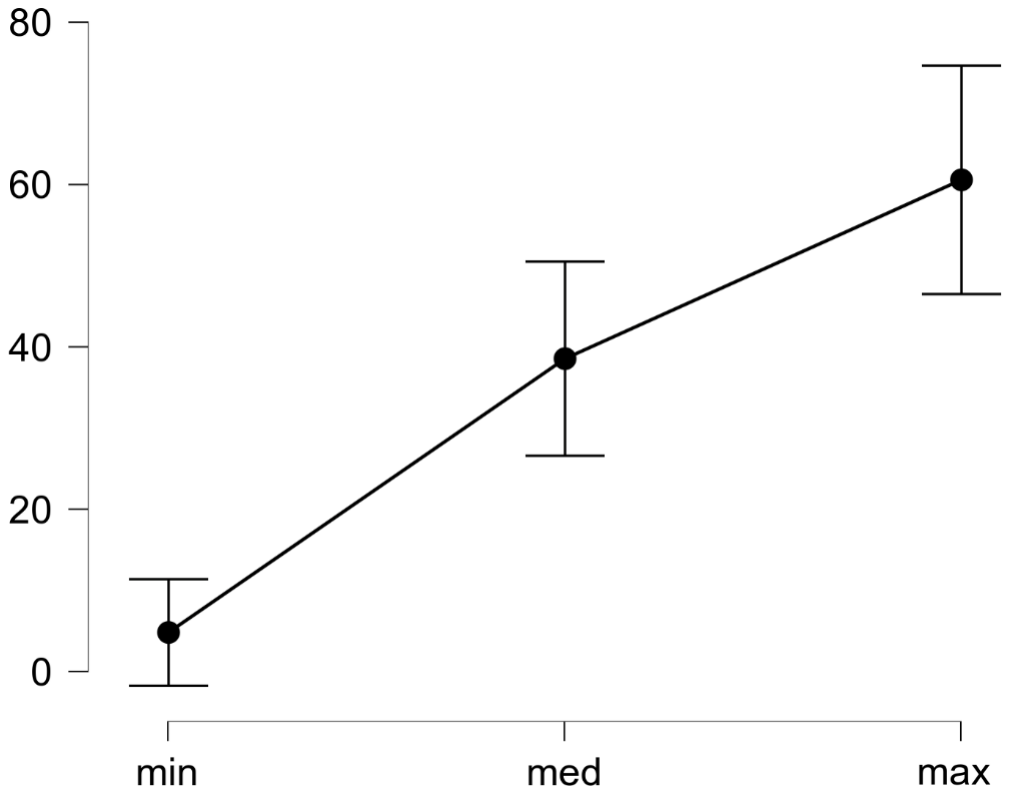
Dependence between the accuracy of recognizing the face emotional expression and the nonlocal contrast modulation amplitude in the areas which have made the stimulus. The abscissa shows the modulation amplitude (see the text explanations).

It was found that in the task of identifying the facial emotional expression the result approximately improves from 5% to 61% with the increase in the modulation amplitude of the total contrast in those fragments from which the stimulus is formed (see Figure 4).

Using ANOVA (JASP software, RRID:SCR_015823) has proved the statistical significance of the dependence obtained (see Table 1). The Levene's test calculation showed a need to use homogeneity corrections.

**Table 1.** Comparison of the accuracy in recognizing face emotional expressions (in the correct answers percentage) for the images with different nonlocal contrast modulation amplitude using ANOVA.

Homogeneity Correction	Cases	Sum of Squares	df	Mean Square	F	p	η ^2^ p
None	amplitude	20497.269	2.000	10248.635	30.332	< .001	0.634
	Residuals	11825.921	35.000	337.883			
Brown-Forsythe	amplitude	20497.269	2.000	10248.635	30.274	< .001	0.634
	Residuals	11825.921	27.843	424.740			
Welch	amplitude	20497.269	2.000	10248.635	37.026	< .001	0.634
	Residuals	11825.921	20.665	572.265			

*Note.* Type III Sum of Squares

The obtained effect is very high (Cohen’s f = 1.3). Post Hoc analysis with the application of Tukey’s test with Bonferroni and Holm’s corrections (see Table 2) also showed that accuracy with which the observers recognize emotions in the faces formed from the areas of different contrast modulation amplitudes, significantly grows with the amplitude increase.

**Table 2.** Post Hoc comparison of the accuracy of recognizing facial expressions for the images with different contrast modulation amplitudes.

		Mean Difference	SE	t	p _tukey_	p _bonf_	p _holm_
max	med	22.035	7.359	2.995	0.014	0.015	0.005
max	min	55.769	7.210	7.735	< .001	< .001	< .001
med	min	33.734	7.359	4.584	< .001	< .001	< .001

*Note.* P-value adjusted for comparing a family of 3

Thus the obtained results have verified our hypothesis stating that the face image areas of the greatest increase of total nonlocal contrast contain information which can be used by the visual system in recognizing emotional expressions.

## Discussion

We used the task of recognizing face emotional expressions in order to demonstrate that the areas of the greatest nonlocal contrast modulation amplitude might possibly be the most informative ones, hence they may be used in categorizing face expressions. Meanwhile the same areas may be revealed by the second-order visual mechanisms.

It should be noted that in recent years there have been publications of a number of model studies where the assessment of the image area aggregate energy is making the basis of the algorithm of segmenting the scenes and selecting objects from the background ( Cheng *et al.*, 2011; Fang *et al.*, 2012; Perazzi *et al.*, 2012). These calculation operations demonstrate really good effectiveness, yet they generally have little in common with the true-life mechanisms of the human visual system.

In our study we too proceeded from the assumption that space heterogeneities of the image energy might contain helpful information. Yet the most important item of our work is that we propose a definite physiological mechanism able of detecting these areas of interest in the image. The developed gradient operator calculating the nonlocal contrast modulation amplitude imitates the functioning of the second-order visual filters with different spatial-frequency tunings. Moreover, we tried to maximally approximate these operators’ parameters to the well-known characteristics of the second-order filters. Thus, for example the spatial frequency (in cycles per “window”) passed from the extracted areas is constant for the “windows” of all the used sizes. This emphasizes the presence of a fixed ratio of the frequency tunings of the first- and second-order filters ( Dakin & Mareschal, 2000; Sutter *et al.*, 1995) and thus ensures the invariance of the description when changing the scale. We have also used a “window” resizing step which provides a change step in the spatial frequency passed by the “windows”, this step equaling 1 octave, which roughly corresponds to the step in the change of the spatial-frequency tuning of pathways in the human visual system ( Wilson & Gelb, 1984). The bandpass of the second-order mechanisms also corresponds to the given bandwidth of our operator and is equal to 1 octave ( Landy & Henry, 2007). We have used the Gaussian envelope in passing the extracted image area, thus imitating the spatial characteristics of the filters at the human visual system input. We have defined that a “window” transmits namely four cycles of the input image. This value is also based on the previously obtained results ( Babenko *et al.*, 2010).

At the same time there were parameters whose optimality remains doubtful to us. So, for example, the number of identified areas grows exponentially in cases where the operator’s size reduces, this chain starting from two “windows”. We have proceeded from the requirement that the total diameter of the identified areas should be equal to the diameter of the whole image. In this case the spatial frequency of the synthesized face may be easily calculated in cycles per image. However, in reality there might be some other number of areas identified at each frequency that is optimal. No doubt, increase in their number will lead to an improved result. Neither did we introduce eccentricity correction since we assumed that in natural conditions saliency maps may also be formed by the human visual system with the use of eye movements. However, the data concerning the time of facial expression perception might indicate that one fixation is sufficient for this ( Liu & Ioannides, 2010; Pourtois *et al.*, 2010; see also reviews George, 2013; Vuilleumier & Pourtois, 2007), although another opinion also exists ( Duncan *et al.*, 2019; Eimer & Holmes, 2007; Eimer *et al.*, 2003; Erthal *et al.*, 2005; Okon-Singer *et al.*, 2007; Pessoa *et al.*, 2002).

Nevertheless, it is impossible to take into account every parameter of the mechanisms providing search for areas of interest in the image and can hardly put into question the conclusion that the information content of the facial image reflecting its emotional expression increases with the growth of the nonlocal contrast amplitude of areas which form this image.

It is also noteworthy that the areas of a maximum nonlocal contrast amplitude can generally be found specifically around the eyes and the mouth (see Figure 1 and Figure 3), i.e. those parts of the face that are considered to be most informative in conveying emotional signals ( Bombari *et al.*, 2013; Eisenbarth & Alpers, 2011; Yu *et al.*, 2018).

## Conclusions

The obtained experimental results have confirmed the hypothesis stating that the image areas of the greatest increase in the nonlocal contrast contain information that contributes to the identification of emotional facial expressions. The second-order visual filters are those mechanisms able to find such information.

We also suppose that the second-order visual filters that highlight the image areas with the highest modulation amplitude of nonlocal contrast are able to attract visual spatial attention; these filters are the windows through which subsequent processing levels receive significant information.

## Data availability

### Underlying data

Open Science Framework: Nonlocal contrast calculated by the second order visual mechanisms and its significance in identifying facial emotions, https://doi.org/10.17605/OSF.IO/5YZGW ( Yavna, 2021).

This project contains the following underlying data:

- emotions.csv –data,
- emotions.jasp – statistics

Data are available under the terms of the Creative Commons Zero "No rights reserved" data waiver (CC0 1.0 Public domain dedication).

## Software availability

Source code available from: https://github.com/dvyavna/2ord_contrast

Archived source code as at time of publication: https://doi.org/10.17605/OSF.IO/5YZGW ( Yavna, 2021).

License: MIT
