# Supplementary material for: Nonlocal contrast calculated by the second order visual mechanisms and its significance in identifying facial emotions
Source: F1000Res. 2023 Aug 29;10:274. Originally published 2021 Apr 6. [Version 2] doi: 10.12688/f1000research.28396.2 (PMC10521119; doi:10.12688/f1000research.28396.2)
Supplement: Peer review history

# Reviewer report: Reviewer response for version 2

**DOI:** 10.5256/f1000research.153731.r201230

**Published:** 2023-09-08

**Authors:** Tina Tong Liu

**Affiliations:** 1 National Institutes of Health, Bethesda, USA

The authors made an effort to revise the manuscript to enhance readability and to promote openness and transparency.

Is the work clearly and accurately presented and does it cite the current literature?

No

If applicable, is the statistical analysis and its interpretation appropriate?

Partly

Are all the source data underlying the results available to ensure full reproducibility?

Partly

Is the study design appropriate and is the work technically sound?

Partly

Are the conclusions drawn adequately supported by the results?

Partly

Are sufficient details of methods and analysis provided to allow replication by others?

Partly

Reviewer Expertise:

Vision, face recognition, emotional processing, primary visual cortex, neuroplasticity

I confirm that I have read this submission and believe that I have an appropriate level of expertise to confirm that it is of an acceptable scientific standard.

# Reviewer report: Reviewer response for version 1

**DOI:** 10.5256/f1000research.31420.r179353

**Published:** 2023-06-20

**Authors:** Tina Tong Liu

**Affiliations:** 1 National Institutes of Health, Bethesda, USA

In this manuscript, the authors aim to identify a mechanism in human visual perception that would be selective for facial features/attributes, which are known to contribute to facial expression recognition. The authors hypothesized that the most informative areas of an image are those characterized by spatial heterogeneity, particularly with nonlocal contrast changes. To test this hypothesis, the authors first developed a software program to create three variants of a single face, each with maximum, minimum, and medium contrast modulation amplitudes. Then, the authors applied the "bubbles" method and ask the participant to judge the emotional expressions of faces. The study found that the greater the contrast modulation amplitude of the areas shaping the face, the more accurately participants were able to identify the emotion, revealing that nonlocal contrast can be a diagnostic feature for facial emotion recognition.  

 While this manuscript addresses a very interesting question, it entails a number of noticeable limitations regarding the 6 review criteria:

**1. Is the work clearly and accurately presented and does it cite the current literature?**

 1.1 I would like to suggest that the authors pay closer attention to citation accuracy.

 For instance, the sentence below could be revised to reflect the appropriate techniques used in the cited studies:

 "Experiments involving a saccadic task (Crouzet *et al.*, 2010; Kirchner & Thorpe, 2006) and registration of event-related potentials (Cauchoix *et al.*, 2014; Dering *et al.*, 2011; Herrmann *et al.*, 2005; Liu *et al.*, 2002; Liu *et al.*, 2009; Pitcher *et al.*, 2007)..."

 Instead of measuring ERP, Liu *et al.* (2002) used MEG, Pitcher *et al.* (2007) used TMS, and Liu *et al.* (2009) specifically measured intracranial field potentials, not event-related potentials. 

 Another example:

 "Indeed, there are specialized mechanisms for finding brightness gradients in the visual system, and these are striate neurons (Hubel & Wiesel, 1962; Hubel & Wiesel, 1968). "

 These two seminal papers focused on the preferences of V1 neurons for orientation, direction of movement, and spatial frequency, not brightness gradients. It is important to note that V1 neurons do not "find" sinusoidal gratings in t he visual scene. The central concept revolves around receptive fields—specific regions in visual space that have an impact on the activity of V1 neurons. 

 1.2 The authors may need to exercise caution when making negative claims that can be readily disproven. 

 For example, "The problem is that the lower levels of the human visual system lack neurons which would be selective to certain facial features" may not hold true based on recent evidence. Recent evidence show that human primary visual cortex is sensitive to emotional expressions of faces (Bo *et al*., 2021 ^1^, Liu *et al.*, 2022 ^2^). 

 Also, "It is clear that the evenly lit areas are of no interest to the visual system." The authors should be aware of the existence of multiple ON and OFF regions within receptive fields, before making this claim. 

 1.3 I would encourage the authors to provide a more balanced view of the literature. 

 The claim that face processing is pre-attentive is not universally accepted. It is crucial for the authors to acknowledge the contradictory nature of prior studies regarding the topic of fast face detection and pre-attentive processing. Please include citation of Pessoa *et al.* (2002) ^3^ for a balanced view of the literature. 

 1.4 The authors should aim to provide a clearer description of the second-order visual processes as proposed by Graham (2011), rather than simply referring to it as a "mechanism." "Mechanism" is a very strong word, meaning the underlying processes, principles, or components that are responsible for this phenomenon. Instead of using the term "second-order visual mechanism," it would be more appropriate to adhere to Graham's (2011) terminology and refer to it as "second-order processes" or "higher-order processes." 

**2. Is the study design appropriate and does the work have academic merit?**

 Based on the information provided, it seems that the stimuli used in the study consisted of 40 initial images of faces and 240 initial images of natural objects. However, it is unclear whether the same task of judging facial expressions was applied to both types of stimuli (faces and natural objects). 

 The authors may need to address the potential limitations regarding the limited number of emotional expressions used in the study. With only two expressions (neutral and happy) presented throughout the experiment, there is a possibility that participants may have identified the diagnostic features associated with these expressions.

 The authors described all the faces have a frontal view, but the sample face using the bubbles procedure in Figure 3 does not have a frontal view. 

**3. Are sufficient details of methods and analysis provided to allow replication by others?**

 The authors may wish to consider breaking down the Method section into subsections to enhance the readability and organization of the manuscript, such as Participants, Study Design, Stimuli, Procedure, etc.

**4. If applicable, is the statistical analysis and its interpretation appropriate?**

 Without detailed information about the specific experimental design, it is challenging to evaluate the appropriateness of using a one-way ANOVA versus a repeated-measures ANOVA.

 There is no standard deviation or error in Figure 2. Simply presenting the mean is not acceptable.

**5. Are all the source data underlying the results available to ensure full reproducibility?**

 I appreciate the authors' efforts to promote openness and transparency, However, to ensure a comprehensive replication of the study, it is important for the authors to provide access to all the processed stimuli, rather than just a limited number of sample images on https://osf.io/5yzgw/files/osfstorage. The emotions.csv file provided in the OSF repository is not sufficient or helpful for replication purposes either. It is recommended that the authors provide PII-stripped (Personally Identifiable Information), anonymized raw data in the OSF registry.

**6. Are the conclusions drawn adequately supported by the results?**

 It is misleading to say "confirmed the hypothesis" or "proved the statistical significance". In the context of null hypothesis testing framework, the stats only allow the authors to reject the null hypothesis. 

 I am not sure if I understand the scope of the conclusions. Is the importance of nonlocal contrast is limited only to facial emotion recognition or if it extends to other aspects such as general face recognition or object recognition in a broader sense?

Is the work clearly and accurately presented and does it cite the current literature?

No

If applicable, is the statistical analysis and its interpretation appropriate?

Partly

Are all the source data underlying the results available to ensure full reproducibility?

Partly

Is the study design appropriate and is the work technically sound?

Partly

Are the conclusions drawn adequately supported by the results?

Partly

Are sufficient details of methods and analysis provided to allow replication by others?

Partly

Reviewer Expertise:

Vision, face recognition, emotional processing, primary visual cortex, neuroplasticity

I confirm that I have read this submission and believe that I have an appropriate level of expertise to state that I do not consider it to be of an acceptable scientific standard, for reasons outlined above.

## References

[1] : Decoding Neural Representations of Affective Scenes in Retinotopic Visual Cortex. *Cereb Cortex* .2021;31(6) : 10.1093/cercor/bhaa411 3047-3063 10.1093/cercor/bhaa411 33594428PMC8107797

[2] : Layer-specific, retinotopically-diffuse modulation in human visual cortex in response to viewing emotionally expressive faces. *Nat Commun* .2022;13(1) : 10.1038/s41467-022-33580-7 6302 10.1038/s41467-022-33580-7 36273204PMC9588045

[3] : Neural processing of emotional faces requires attention. *Proc Natl Acad Sci U S A* .2002;99(17) : 10.1073/pnas.172403899 11458-63 10.1073/pnas.172403899 12177449PMC123278

# Response

**Published:** 2023-07-28

**Authors:** Denis Yavna

**Affiliations:** Southern Federal University, Russian Federation

Dear Reviewer

 We sincerely thank you for your attention to our manuscript and your valuable comments. We tried to take into account all of them and made changes to the text.

 Let me also make some comments.

*1.1 I would like to suggest that the authors pay closer attention to citation accuracy.*

 Thank you, your comments have been taken into account. The only thing we have left unchanged is the reference (Hubel & Wiesel, 1962).

 You are correct that "These two seminal papers focused on the preferences of V1 neurons for orientation, direction of movement, and spatial frequency..." However, it is in the article by Hubel & Wiesel (1962) that the authors first state: “The most effective stimulus configurations, dictated by the spatial arrangements of excitatory and inhibitory regions, were long narrow rectangles of light (slits), straight-line borders between areas of different brightness (edges), and dark rectangular bars against a light background". This is a direct indication that striate neurons respond most strongly to brightness gradients (bands or edges).

 1.2 *The authors may need to exercise caution when making negative claims that can be readily disproven.*

 We have included citations of relevant papers in the text. However, we are not sure that we have the right to insert citations of papers published after our manuscript was submitted. We also clarified what we mean by "lower levels of the human visual system" in the text.

 As for the On- and Off-reactions of the visual neurons, of course they take place. However, in the context of the problem of saliency, these reactions are not considered. At the same time, turning on or off the light stimulus can also attract the observer's attention. Therefore, we have made an appropriate addition to the text.

 1.3 *I would encourage the authors to provide a more balanced view of the literature.*

 Indeed, "The claim that face processing is pre-attentive is not universally accepted." We have added links.

 1. *4 The authors should aim to provide a clearer description of the second-order visual processes*

 We have included in the text a more detailed description of the second-order filter model.

 As for the term "mechanism", you correct: "Mechanism" is a very strong word." Where possible, we have replaced the word “mechanism”.

***2. Is the study design appropriate and does the work have academic merit?***

*А* *) it is unclear whether the same task of judging facial expressions was applied to both types of stimuli (faces and natural objects).*

 We have included in the text an explanation of how the subjects' task was formulated.

*B) there is a possibility that participants may have identified the diagnostic features associated with these expressions.*

 We agree with you that such a possibility existed. Therefore, we tried to minimize it. To do this, the subjects had to categorize all presented stimuli (not just faces). Faces appeared much less frequently than non-faces and differed, in addition to expression, in sex, race, and frequency content. The subjects were not warned in advance which facial expressions would be used. They themselves had to determine the facial expression if they detected a face in a series of other stimuli.

*C) The authors described all the faces have a frontal view, but the sample face using the bubbles procedure in Figure 3 does not have a frontal view.*

 We used face images from a database that labeled them as frontal. However, there were some variations of the angle. We considered this to be a positive factor, since it introduced additional variability into the incentives.

***3. Are sufficient details of methods and analysis provided to allow replication by others?***

 Based on your recommendation, we have structured the "Method" section into subsections.

***4. If applicable, is the statistical analysis and its interpretation appropriate?***

 We have made appropriate corrections to the text.

***5. Are all the source data underlying the results available to ensure full reproducibility?***

 Based on your recommendation, we have provided access to all source data.

***6. Are the conclusions drawn adequately supported by the results?***

 We have made corrections in accordance with your comments.

 Once again, we sincerely thank you for your attention to our manuscript and for your valuable comments.

# Reviewer report: Reviewer response for version 1

**DOI:** 10.5256/f1000research.31420.r82785

**Published:** 2021-05-06

**Authors:** Yuri E. Shelepin

**Affiliations:** 1 Pavlov Institute of Physiology, Russian Academy of Sciences, St. Petersburg, Russian Federation

This article solves the problem of the most informative areas of the face images. Authors argue that these areas are in the greatest increase of non-local contrast. A model of the second-order visual mechanism is used, with the help of which the image areas with the highest, lowest and intermediate amplitude of the total contrast modulation are extracted. The answers of the observers in the task of distinguishing the emotional expressions in face images created from areas with different modulation amplitudes are analyzed.

 Evidence is given that the second-order visual filters can play the role of preattentive operators, highlighting the most informative image areas. Overall, the finding of the current study is important and interesting, and analysis is reliable. However, I would like to highlight two problems:

1. The second-order visual mechanisms should be described in the Introduction in more detail, since the journal has a broader audience.
2. The authors used the task of distinguishing facial expressions and obtained a result confirming the informational significance of the image areas with the highest total brightness contrast. However, different information is useful for different visual tasks. At the same time, are the preattentive mechanisms specific to the visual task? Is the proposed algorithm for finding the interest areas effective in the task of discriminating emotions and will it be equally effective in other visual tasks? It would be useful to address this issue in the Discussion.

Is the work clearly and accurately presented and does it cite the current literature?

Partly

If applicable, is the statistical analysis and its interpretation appropriate?

Yes

Are all the source data underlying the results available to ensure full reproducibility?

Yes

Is the study design appropriate and is the work technically sound?

Yes

Are the conclusions drawn adequately supported by the results?

Yes

Are sufficient details of methods and analysis provided to allow replication by others?

Yes

Reviewer Expertise:

Vision perception and pattern recognition

I confirm that I have read this submission and believe that I have an appropriate level of expertise to confirm that it is of an acceptable scientific standard.

# Response

**Published:** 2021-05-12

**Authors:** Denis Yavna

**Affiliations:** Southern Federal University, Russian Federation

Thank you very much for your attention to our study and its positive assessment. We will add necessary information to the Introduction and Discussion.
